# The Study of Approaches to Modeling Oxidative Stress in Male Wistar Rats: The Comparative Analysis of Diet-Induced, Chemically Induced, and Physiologically Induced Models

**DOI:** 10.3390/ijms26146872

**Published:** 2025-07-17

**Authors:** Yuliya Sidorova, Nikita Petrov, Nadezhda Biryulina, Ilya Sokolov, Anastasiya Balakina, Nikita Trusov, Alla Kochetkova

**Affiliations:** Federal Research Centre of Nutrition and Biotechnology, 109240 Moscow, Russia; petrov-nikita-y@mail.ru (N.P.); biryulina_nadezhda@mail.ru (N.B.); sokolov.iliya1993@gmail.com (I.S.); balakina.a.s@yandex.ru (A.B.); nikkitosu@yandex.ru (N.T.); kochetkova@ion.ru (A.K.)

**Keywords:** oxidative stress, biological model, antioxidant status, high-cholesterol diet, immobilization, CCl_4_

## Abstract

Oxidative stress can be caused by bad nutrition, psychoemotional stress, or other external influences in everyday life. The aim of this study is to develop and verify biological models using male Wistar rats that allow reproducing oxidative stress in vivo, in particular: food (diet with high cholesterol and fructose content), medical (injection of CCl_4_), and physiological (immobilization stress). Diet with 1% cholesterol and fructose had the greatest impact on the antioxidant status of animals: a significant increase in serum malondialdehyde (by 1.8 times) and superoxide dismutase (by 2.4 times) as well as a significant decrease in the Cat gene expression by 35% were shown. The immobilization led only to a significant decrease in serum lipid peroxides by 29%. A single intraperitoneal administration of CCl_4_ was accompanied by a significant increase in the blood lipid peroxides (by 1.3 times) and catalase (by 1.6 times), as well as a significant decrease in the *Cat* mRNA by 33% and *Gpx1* by 48%. The obtained data can be used to study the effectiveness of drugs, biologically active food supplements and functional nutrition in vivo.

## 1. Introduction

Oxidative stress occurs as a result of imbalanced production of reactive oxygen species (ROS). Excessive formation of ROS may lead to oxidative damage of molecules involved in aging and development of neurodegenerative and cardiovascular diseases. To increase a human organism’s resistance to oxidative stress, it is necessary to provide it with antioxidants. It is necessary to select an appropriate biomodel that allows reproducing oxidative stress in laboratory rodents and obtaining reliable results.

Oxidative stress may be caused by bad nutrition, psycho-emotional stress or other external influences in everyday life. Excessive consumption of diets high in fat, especially saturated fatty acids, fried and smoked meats, baked goods, juices, and soda (sources of cholesterol and simple sugars) leads to the development of hyperlipidemia and obesity. The subsequent accumulation of adipose tissue in the body induces the development of oxidative stress, endothelial dysfunction, and excessive synthesis of adipokines, leading to other complications [1,2]. To simulate such conditions in vivo, diets with a high content of fat (45–60% fat by calories), easily digestible carbohydrates, and cholesterol are widely used. The so-called “Cafeteria” diets, high in fat and sugar, simulate modern human obesogenic diets [3,4,5,6]. However, due to heterogeneity and a lack of standardization, this model is less effective. Obesity and associated diseases in animals develop slowly and generally replicate the corresponding human pathology when fat accumulates slowly, mainly due to excess energy intake and insufficient energy expenditure. The use of a high-cholesterol diet efficiently leads to conditions characterized by the accumulation of prooxidants and lipid peroxidation products [7].

Besides the diet-induced models, drug-based approaches using various chemical compounds have traditionally been used to model oxidative stress. Paracetamol (acetaminophen) [8], lipopolysaccharide [9], and carbon tetrachloride are most commonly used in in vivo studies. Paracetamol, which is a commonly used antipyretic and analgesic drug, is safe and effective at therapeutic doses. The main limitation of this model is the use of high doses to induce mitochondrial dysfunction, which leads to overproduction of ROS and further liver damage [10]. Lipopolysaccharide is a bacterial pathogen that imbalances oxidative stress status and immune system responses mainly through the overproduction of pro-inflammatory cytokines [11]. Carbon tetrachloride (CCl_4_) is a well-known toxin associated with liver damage, causing oxidative stress and damage to cellular components [12,13,14]. It is a potent prooxidant agent that has been widely used in recent years to induce hepatotoxicity [15] and oxidative stress [16]. CCl_4_ administration causes oxidative damage through the formation of free radicals and enhances lipid peroxidation [16]. Although carbon tetrachloride administration to animals is one of the most widely used in vivo models of oxidative stress/lipid peroxidation, it has several limitations. The induced oxidative stress is a consequence of poisoning and acute failure of vital organs, especially the liver [17]. The toxicity of CCl_4_ depends on the dose and exposure time. At low doses, temporary effects prevail: disorders in lipid homeostasis, cytokine release, and apoptosis. At higher doses or longer exposure, fatty degeneration, fibrosis, cirrhosis, and even cancer may occur. At acute toxic doses irreversible liver injuries take place [18].

As an alternative for modeling oxidative stress, some studies use stress exposure [19,20]. Increased metabolic rate causes excessive formation of free radicals [21], in particular reactive oxygen species, accompanied by functional and structural damage to cells and tissues. For example, acute exercise on a treadmill causes a significant release of pro-inflammatory cytokines and free radicals from activated leukocytes and leads to muscle damage and tissue injury. Moreover, during prolonged exercise, oxygen consumption increases and produces reactive oxygen species (ROS). However, this method has limitations, like the necessity for preliminary training of animals and possible injuries during the test [22]. The forced swim test (FST) has been reported to increase rodent oxidative stress in the brain as well as tissue degeneration and oxidative stress in peripheral tissues, including the liver, which is regulated by the brain [23]. The necessity to warm and dry animals and potent injuries are also limitations of this method. To study physiological reactions caused by a state of chronic psychoemotional stress, a model of immobilization is used [24]. Stress caused by immobilization leads to behavioral changes such as anxiety, depression, anorexia, and decreased activity, similar to human reactions to crowding, frustration, and tension [25]. The model is easily reproduced and used to simulate acute or chronic stress [26].

The aim of the present study is to conduct a comprehensive comparative analysis of three distinct models of oxidative stress in male Wistar rats: dietary-induced stress, chemically induced stress via intraperitoneal injection of CCl_4_, and physiologically induced stress through immobilization. This research seeks to evaluate and compare the extent and nature of oxidative stress elicited by each method, with particular focus on a panel of biochemical markers, including lipid peroxidation markers (LPO, MDA), antioxidant enzymes (SOD, catalase, glutathione peroxidaze), and antioxidant genes liver expression (*Sod1*, *Gpx1*, *Cat*, *Nqo1*, and *Hmox1*). This investigation seeks to elucidate the differential effects and mechanisms underlying each model, thereby contributing to a more nuanced understanding of oxidative stress responses in preclinical research.

## 2. Results

### 2.1. Fatty Acid Composition

Table 1 presents the results of determining the fatty acid composition of sunflower and soybean oils used in diets.

Soybean oil is a source of linoleic, oleic, palmitic, α-linolenic, and stearic fatty acids. Sunflower oil is a source of linoleic, oleic, palmitic, and stearic fatty acids. Thus, the main difference in the fatty acid composition of soybean oil from sunflower oil is the content of α-linolenic ω-3 fatty acid.

### 2.2. In Vivo Study Results

**Figure 1:**
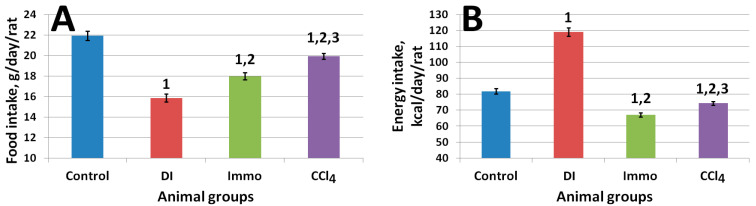
(**A**) Average food intake, g/day/rat (η^2^ = 0.40); (**B**) average energy intake, kcal/day/rat (η^2^ = 0.71). ^1^ differences are significant against control group; ^2^ differences are significant against DI group; ^3^ differences are significant against Immo group; *p* < 0.01.

The general condition of all animals in terms of appearance, fur quality, food and water consumption, and behavior during daily examination was satisfactory. Figure 1 shows the average cumulative feed and calorie consumption of animals throughout the experiment.

Animals of the diet-induced (DI) group consumed significantly less food compared to animals of the control, Immo, and CCl_4_ groups. However, in terms of caloric content, these animals, on the contrary, received significantly more energy than the others, since in addition to feed, they consumed an average of 50 ± 3 mL of a 30% fructose solution.

Animals of groups Immo and CCl_4_ consumed significantly less food and energy compared to control animals, while animals of group Immo consumed less food compared to animals of group CCl_4_. Thus, immobilization stress led to a significant decrease in animals’ appetite compared to all other groups. Replacing the fat component in the diet, namely soybean oil, with sunflower oil also led to a significant decrease in appetite.

**Figure 2:**
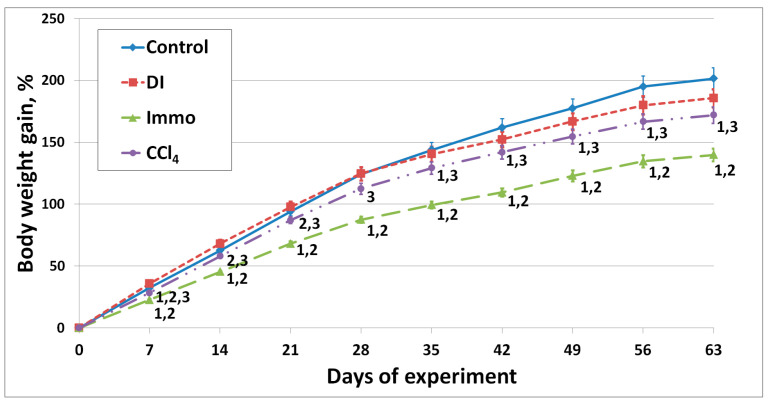
Body weight gain of experimental animals, %. ^1^ differences are significant against control group; ^2^ differences are significant against DI group; ^3^ differences are significant against Immo group; *p* < 0.05.

Figure 2 shows the body weight gain of animals throughout the experiment.

Starting from the 7th day, a significant lag in the body weight gain of Immo group animals was observed compared to the control, DI, and CCl_4_ animals. The difference remained significant throughout the experiment. The body weight gain of CCl_4_ animals, starting from day 35, was significantly lower compared to the control until the end of the experiment. The obtained result is consistent with the food intake data. The body weight gain of DI animals did not differ significantly from the control, despite higher calorie consumption.

**Figure 3:**
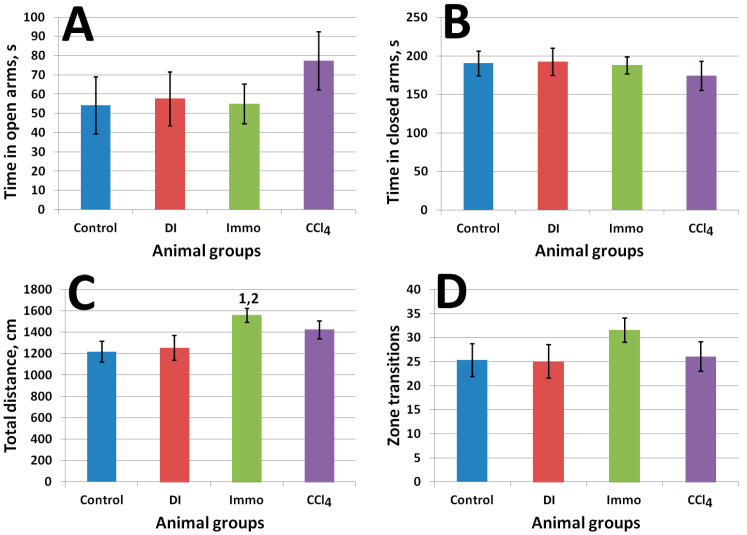
Elevated plus maze test results. ^1^ differences are significant against control group; ^2^ differences are significant against DI group; *p* < 0.05. (**A**) Time in open arms, s (η^2^ = 0.04); (**B**) time in closed arms, s (η^2^ = 0.02); (**C**) total distance (η^2^ = 0.170); (**D**) zone transition number (η^2^ = 0.06).

Figure 3 shows the results of the elevated plus maze test.

Animals of group Immo showed more activity, reflected by a significantly greater distance traveled, compared to the control (*p* = 0.02) and DI (*p* = 0.03) animals. The obtained result indicates the influence of immobilization on the component of exploratory and motor activity, characterized by the distance traveled, without changing the degree of anxiety.

Table 2 shows the body composition of experimental animals.

As can be seen from the presented data, the body fat mass of Immo animals was significantly lower compared to control animals and DI animals, while their lean mass, on the contrary, was significantly higher compared to these groups. Also, the total water content in the body of these animals was significantly higher compared to control and DI animals.

Table 3 shows the results of biochemistry blood test of experimental animals.

Animals of the DI group had a significant increase in total protein against the background of a significant increase in globulins compared to control animals. Consumption of exogenous cholesterol and fructose resulted in a significant increase in the blood cholesterol and LDL levels compared to control animals, while the triglyceride level was significantly lower. A significant increase in the serum ALT level and a decrease in serum urea were revealed compared to the control group. According to mineral metabolism, a reliable decrease in blood magnesium level of these animals was observed. The level of serum glucose and complete blood glucose in these animals was significantly higher compared to all groups, no changes in the glycated hemoglobin level were found.

In animals of Immo group with normal levels of total cholesterol, a significant decrease in triglyceride levels and an increase in LDL levels were found compared to animals of the control group. Also, animals of this group showed a significantly higher level of ALT compared to control animals.

A significantly lower concentration of serum cholesterol and triglycerides was found in animals of CCl_4_ group compared to the control group. The ALT level was also significantly higher compared to animals of the control group. The animals of this experimental group had the lowest serum glucose: the differences are significant compared to the control group, while no difference was found in whole blood glucose. The level of glycated hemoglobin also differed significantly and was lower than in control animals.

Table 4 presents the biochemical parameters determined in the liver of animals.

Animals of the DI group had the highest relative liver weight and the highest liver fat content; the differences were significant with all groups. Cholesterol and triglyceride levels were significantly higher compared to those in the control group.

Animals of the Immo group at the end of the experiment had significantly lower body weight, relative liver weight, and liver fat content compared to the control group. Moreover, the levels of cholesterol and triglycerides did not differ significantly from those in the control group.

At the end of the experiment, the body weight of animals in the CCl_4_ group was significantly lower compared to the control group. The level of cholesterol in the liver of these animals was significantly higher compared to the control group, and the level of triglycerides was significantly lower than the control group.

Biochemical parameters of blood serum determined by ELISA are shown in Table 5.

A significant accumulation of MDA, a product of lipid peroxidation, was noted in DI animals. Also, SOD activation in the blood of these animals was shown; the accumulation of SOD levels in their blood was significantly different compared to control animals that received a standard diet. Daily forced immobilization resulted in a significant decrease in blood lipid peroxides of animals compared to the control group. As a result of a single intraperitoneal injection of CCl_4_ a significant accumulation of lipid peroxide levels and activation of catalase were noted in experimental animals compared to control.

**Figure 4:**
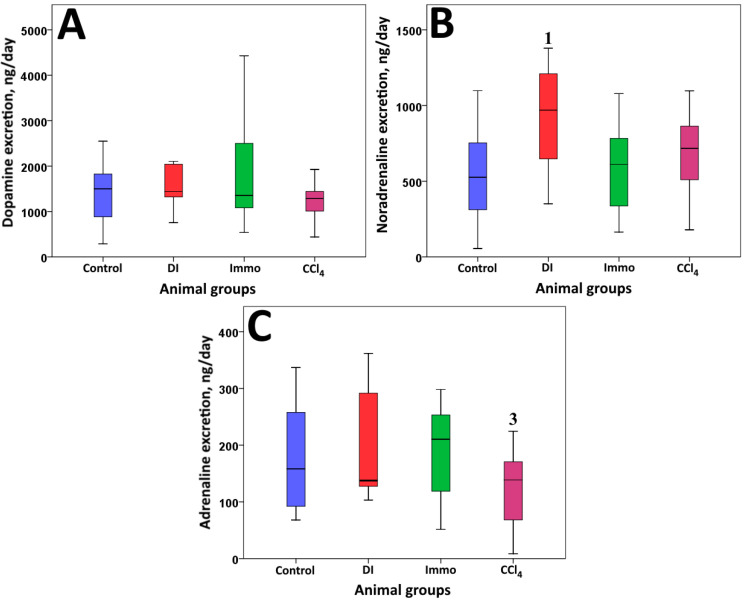
Catecholamine daily urine excretion. (**A**) Dopamine, ng/day; (**B**) noradrenaline, ng/day; (**C**) adrenaline, ng/day. ^1^ differences are significant compared to control group; ^3^ differences are significant compared to Immo group; *p* < 0.05. η^2^ values are: (**A**) 0.12; (**B**) 0.18; (**C**) 0.11.

Figure 4 shows the results reflecting the excretion of catecholamines in urine: dopamine, noradrenaline, and adrenaline.

A significant increase in noradrenaline (*p* = 0.03) was found in the urine of DI animals compared to control animals, while there were no significant differences in other indicators. The obtained result may be a consequence of activation of the sympathicoadrenal system in response to the toxic effects of cholesterol and fructose. Animals of the CCl_4_ group had a significant decrease (*p* = 0.04) in the adrenaline level compared to animals of the Immo group.

As shown by the PCR analysis (Table 6), a significant decrease in the expression of the *Cat* gene by 35% (*p* = 0.013) was found in animals of the DI group, relative to the control level, as well as a moderate decrease, at the level of trends, in the expression of the *Gpx1* gene by 20% (*p* = 0.059) and *Sod1* gene by 21% (*p* = 0.092) compared to the control group. At the same time, a high-calorie diet had no statistically significant effect on the expression of the transcription factor Nrf2 and Nf2-mediated antioxidant defense enzyme genes *Hmox1* and *Nqo1*. No statistically significant changes in the expression of the studied genes were detected in animals of the Immo group compared to the control. A single intraperitoneal injection of CCl_4_ solution resulted in a significant decrease in the amount of *Cat* mRNA by 33% (*p* = 0.02) and *Gpx1* mRNA by 48% (*p* = 0.003) compared to the control. Moreover, a significant 5-fold increase in the expression of the *Nqo1* gene (*p* = 0.004) was found in the liver of these rats compared to the control group.

## 3. Discussion

Rats treated with a diet high in cholesterol and fructose consumed significantly less food, despite receiving almost 1.5 times more energy due to fructose. However, the body weight gain, as well as the fat/lean mass ratio in these animals, did not differ significantly from the control. In our case, consumption of this diet did not lead to the development of obesity but only affected the metabolic processes.

Animals subjected to forced immobilization consumed significantly less food and energy (by 18%), which led to a significant reduction in body weight gain compared to all groups of animals. The fat mass in the body composition of these animals was also significantly lower. The immobilization model has a serious impact on the psycho-emotional state of animals. That is accompanied by loss of appetite, leading to an exhaustion of the body, a decrease in organ weight and a change in a number of biochemical parameters [27]. The ratio of both total and free water in the body of these animals was significantly higher compared to the control. The obtained result is consistent with the data presented in the study [28], where immobilization stress led to a decrease in daily diuresis of experimental rats, which led to water retention in the animals’ bodies.

A decrease in both appetite and body weight gain was detected in animals of CCl_4_ group. The obtained result is explained by differences in the diet: the animals of this group received a diet with the inclusion of sunflower oil, while control animals received soybean oil, a source of ω-3 PUFA, in particular α-linolenic acid (Table 1). The study [29] also showed that the introduction of ω-3 PUFAs into the diet of rats promotes the appetite and, accordingly, food consumption.

According to numerous studies, long-term consumption of a high-cholesterol diet causes lipid metabolism disorders and oxidative stress [30,31,32]. Feeding animals a diet containing 1% cholesterol for 9 weeks resulted in increased serum total cholesterol and LDL, indicating the development of hypercholesterolemia and dyslipidemia. This is also confirmed by extreme liver fat accumulation and increased liver cholesterol and triglyceride levels. Fructose consumption resulted in a significant increase in blood glucose level. A significant increase (by 60%) in the relative liver weight was also noted compared to the control group. The obtained results suggest that such a diet leads to disorders in internal organs and metabolic systems.

Rats subjected to chronic immobilization stress had decreased relative liver weight and liver fat content. The blood level of triglycerides was also significantly lower compared to control animals. As mentioned above, severe psycho-emotional stress leads to exhaustion of the body and a decrease in organ weight.

A single intraperitoneal injection of CCl_4_ had a strong toxic effect on rats’ metabolic processes. We observed a significant decrease in the blood levels of cholesterol, triglycerides, glucose, and glycated hemoglobin compared to the control. Similar results were obtained in the work [33]. Liver intoxication with CCl_4_ leads to a decrease in glycogen stores, inhibiting gluconeogenesis and decreasing blood glucose level [34]. The content of glycated hemoglobin characterizes the average blood glucose level over the last 60 days, which corresponds to the average lifespan of erythrocytes in rats [35]. The reaction of glucose binding to hemoglobin is irreversible; therefore, glycated hemoglobin circulates in the blood until the death of the bound erythrocyte [36].

Excessive accumulation of reactive oxygen species (ROS) in the liver leads to injury of hepatocyte membranes. The process of lipid peroxidation (LPO) is activated there with the production of lipid peroxides and MDA [37,38].

The blood level of lipid hydroperoxides and MDA reflects the degree of cell damage due to lipid peroxidation processes [39]. A significantly higher level of blood MDA in the animals treated with a diet high in cholesterol and fructose indicates the activation of lipid peroxidation processes. Similar results were obtained in the works [40,41,42,43], where the consumption of high-cholesterol diets by laboratory rodents (rats, mice, hamsters, rabbits) led to an increase in the serum levels of MDA and lipid peroxides. We also revealed a significant increase in the blood levels of lipid peroxides in animals that received a single injection of CCl_4_, compared to the control. CCl_4_ is a potent hepatotoxin and is rapidly transformed by cytochrome P450 in the presence of oxygen into metabolites, that damage polyunsaturated fatty acids of membrane phospholipids, leading to the formation of peroxides and the initiation of lipid peroxidation [17,37,44].

Activation of lipid peroxidation provokes a response from the antioxidant defense system. A significant increase in the blood level of SOD was found in animals fed a high-cholesterol diet with fructose, compared to control animals. The obtained data show the activation of the antioxidant defense system since the synthesis of SOD increases in response to lipid peroxidation. The study [30] also noted an increase in the levels of antioxidant defense enzymes—SOD, glutathione reductase, and xanthine oxidase—in the blood of male Wistar rats fed a high-fat, high-fructose diet for 18 months. The activation of body’s compensatory mechanisms is also confirmed by a significant increase in the blood total protein of animals, which is the marker of liver synthesizing activity [12]. Rats subjected to a single injection of CCl_4_ showed significantly higher levels of catalase, which also indicates the activation of the antioxidant defense system in these animals. Catalase is one of the main enzymes that prevent cellular accumulation of hydrogen peroxide, which promotes the formation of the hydroxyl radical. The level of catalase increases in response to an increase in the level of hydrogen peroxide.

AST and ALT are deposited mainly in the heart and liver. An increase in their blood level may indicate a disruption in the functioning and integrity of hepatocytes [45]. The results showed a significant increase in the level of blood ALT in the animals of all experimental groups. Blood AST level was also significantly increased in animals subjected to forced immobilization. A significant increase in the level of blood transaminases and a more than 2.5-fold increase in the AST over ALT confirms the exhaustive effect of immobilization on the rats [46]. The obtained data are also consistent with the results of the work [12], where intraperitoneal administration of CCl_4_ to mice and rats led to a significant increase in the levels of serum transaminases of the animals. We also noted a visible (but not significant, *p* = 0.124) increase in the blood AST level in animals receiving a diet high in cholesterol and fructose.

Catecholamines are the major neurotransmitters that mediate various central nervous system functions such as locomotor activity, cognition, emotion, memory, and endocrine regulation. These hormones have the ability to reduce oxidative stress by scavenging free radicals and binding metal ions. The presence of a catechol fragment in the structure determines the activity of catecholamines in removing free radicals [47]. A significant increase in the daily excretion of norepinephrine was found in animals fed a diet high in cholesterol and fructose compared to control animals. This is a consequence of increased activity of the sympathoadenal system in response to the toxic effects of exogenous cholesterol on the animal body [48]. Increased levels of catecholamines in the body may also be associated with increased production of reactive oxygen species [49].

On the other hand, the high reactivity of catecholamines to free radicals also means that these molecules will break down over time, losing their function and forming potentially toxic metabolites [47]. In animals that received a single injection of CCl_4_, a tendency toward decreased adrenaline excretion was noted (*p* = 0.222 relative to the control group and *p* = 0.106 relative to the DI group), and the differences with animals subjected to immobilization stress were significant (*p* < 0.05).

As the level of ROS increases, protective antioxidant mechanisms activate to degrade them. Our study showed a decrease in the expression of key antioxidant enzyme genes *Cat*, *Gpx1* and *Sod1* in the liver of rats of the DI and CCl_4_ groups. This indicates a weakening of the antioxidant defense system and the development of oxidative stress in these groups. Thus, a single intraperitoneal injection of CCl_4_ resulted in decreased activity of the GSH, SOD, and CAT [50]. In similar studies on male Wistar rats, a single administration of CCl_4_ resulted in a significant decrease in the activity of SOD, CAT, and GSH and the expression of the *Sod1* gene. The authors attribute this decrease to the depletion of antioxidant enzymes as a result of oxidative stress caused by the administration of CCl_4_ [51].

The Nrf2/Keap1/ARE signaling pathway plays a key role in protecting the cell from oxidative stress. The antioxidant and phase II detoxification enzymes regulated by this signaling pathway can eliminate ROS and suppress the biotransformation of xenobiotics. Activated transcription factor Nrf2 can induce expression of protective enzyme genes such as *Hmox1* and *Nqo1* to resist oxidative stress damage [52].

A single intraperitoneal administration of CCl_4_ resulted in a moderate decrease in the expression of *Nrf2* and *Hmox1* genes and a 5-fold increase in the expression of *Nqo1*. A similar study in male Sprague-Dawley rats injected intraperitoneally with CCl_4_ showed a decrease in *Nrf2*, *Hmox1*, and *Nqo1* mRNA levels in rat liver [53]. In the other study, oral administration of CCl_4_ to male Sprague-Dawley rats for 6 weeks caused liver fibrosis, as well as a three-fold increase in the liver expression of NQO1 protein, while the amount of hemeoxygenase-1 protein did not change compared to the control [54]. The administration of CCl_4_ was also accompanied by a significant increase in the expression of NQO1 protein in the rat liver. The authors suggest that increased NQO1 expression occurs in response to ROS generation caused by inflammation or xenobiotic transformation. NQO1 is associated with cellular redox balance, protecting the cell from free radicals inhibiting the one-electron reduction of quinines [55].

### Limitations of the Study

The main limitation of this study is the difference in the fat component of the groups’ diets. The standard AIN-93 diet contains soybean oil in the fat component. Soybean oil is a source of ω-3 PUFAs, whose consumption can prevent the development of oxidative stress. Accordingly, the diets of the experimental groups included sunflower oil, which does not contain ω-3/6 PUFAs. Moreover, the use of sunflower oil provides a closer approximation to the average human diet, which often includes sunflower oil as the most accessible product. Also, the combination of sunflower oil, margarine, cholesterol and fructose in our study is used to simulate an unhealthy diet with a high proportion of saturated fats and easily digestible carbohydrates.

The use of only one dose of CCl_4_ in a single administration can also be considered as a limitation of the presented study. According to literature data, the toxic effect of CCl_4_ depends on the dose and exposure time, from weaker reversible effects to severe irreversible injuries. We have chosen a single administration so that the toxic effect of CCl_4_ would be reversible. Although studies using other doses, as well as chronic exposure to this substance, are of scientific interest and have been published repeatedly. Evaluation of the chronic effects of CCl_4_ on the antioxidant system and internal organs of animals may become the subject of our further studies.

## 4. Materials and Methods

### 4.1. Experimental Animals

The experiment was conducted using 50 Wistar male rats (age 4 weeks) with an initial body weight of 100 ± 2 g. Animal studies were carried out in accordance with standard principles described in “Guide for the Care and Use of Laboratory Animals” [56] and approved by the Local Ethics Committee (Protocol No. 8 dated 6 September 2024). Animals were kept under controlled environmental conditions (temperature 20–24 °C, relative humidity 30–60%, 12 h lighting cycle).

The open field (OF) test was used to divide animals into groups [57].

**Figure 5:**
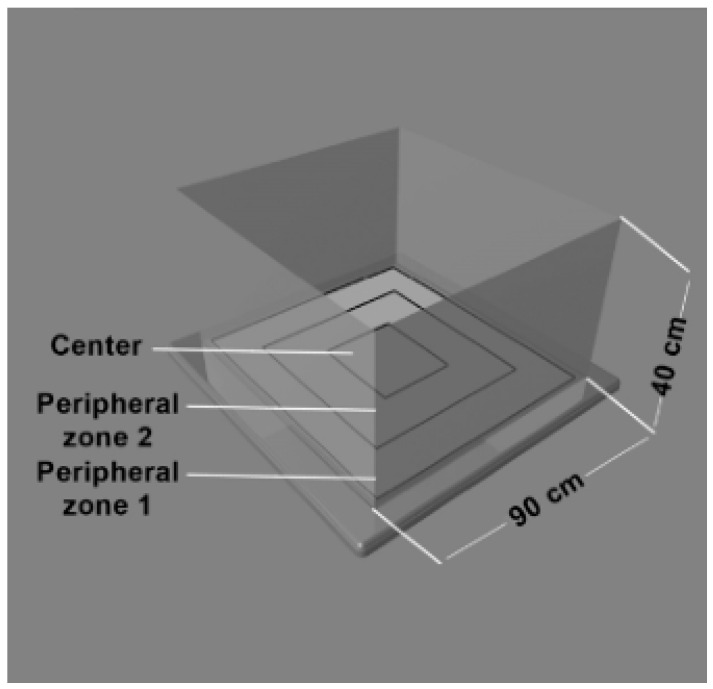
Open field box.

The open field box was a black square arena (90 cm × 90 cm) surrounded by gray walls (40 cm) and divided into one central zone and two peripheral zones (Figure 5).

The animals were placed in the corner and allowed to explore the box for 3 min. To evaluate the behavior, the following parameters were used: horizontal (number of transitions between zones, total distance), the latency of the first move, the latency of the first entry into the central zone, time spent in different zones of the field. The test was conducted during the period of the animals’ minimal activity (between 10 a.m. and 3 p.m.).

The movement of animals was recorded using the Smart 3.0.04 video-tracking system (Panlab, Barcelona, Spain).

### 4.2. Determination of Fatty Acid Composition of Oil Samples (Sunflower and Soybean)

About 10 mg of the lipophilic fraction of each sample was added to screw-capped tubes with a liner. Further, 2.0 mL of methanol (chemically pure, Aquametry, Moscow, Russia), 20 µL of hexane, and 20 µL of acetyl chloride (Acros organics, Geel, Belgium) were added to the samples. The test tubes were tightly closed with lids and, after brief vigorous shaking, were placed in a drying oven for 1 h at 80 °C to methylate the fatty acids. After cooling the samples to room temperature, 2.5 mL of hexane and 100 µL of deionized water were added to them, and they were vigorously mixed on a vortex shaker for about 10 s. After separation, 1.0 mL of the upper hexane layer containing methyl esters was transferred to vials for gas chromatographic analysis.

#### Gas Chromatography Conditions with Flame Ionization Detector

Sample injection volume 1 µL, flow split mode 50:1, carrier gas hydrogen, flow rate 1.65 mL/min. Injector temperature 200 °C, detector temperature 240 °C. Separation conditions: initial temperature 100 °C (isotherm for 3 min), then increase at a rate of 3 °C/min to 200 °C, isotherm for 3 min, then increase at a rate of 3 °C/min to 240 °C, isotherm for 6 min. Data collection and processing were carried out using Agilent ChemStation Rev.B.04.03 and Microsoft Excel 2007 software, respectively. The ratio of fatty acids was determined by the internal normalization method.

### 4.3. Experimental Design

Open Field testing of animals was conducted after seven days acclimatization prior to the start of the experiment. Animals were divided into 4 groups (n = 12) based on body weight and OF test results: control, diet-Induced (DI), Immo, and CCl_4_. The groups were formed to achieve the same average weight and the same average OF activity of animals between the groups (Table 7).

Animals of all groups received a standard semi-synthetic diet according to AIN93G with some modifications [58] and water ad libitum during the 63 days of the experiment.

**Figure 6:**
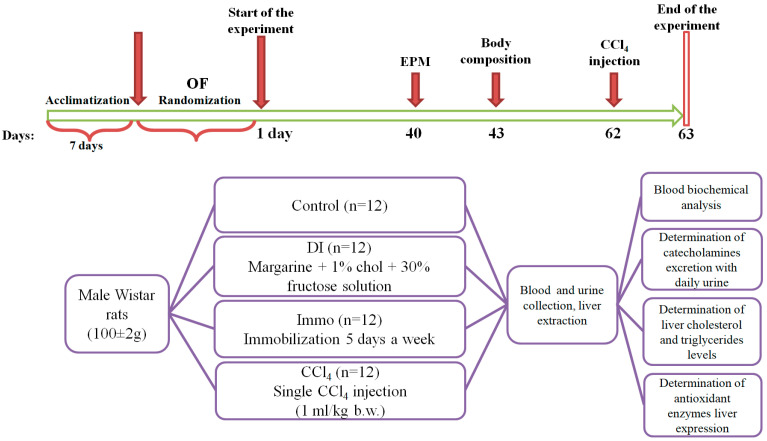
Design of the experiment.

The experimental design is presented in Figure 6.

Animals of the control group received a standard diet (lard and soybean oil as fat components, 375 ± 3 kcal). Animals of group DI received a standard semi-synthetic diet (lard and margarine as fat components) with the addition of 1% cholesterol (375 ± 3 kcal). The animals received a 30% fructose solution as their only source of drink. Animals of group Immo received a standard semi-synthetic diet (lard and sunflower oil as fat components, 375 ± 3 kcal). The fatty acid composition of oils used in diets is shown in Table 1. These animals were subjected to 30 min forced immobilization in plastic restrainers (Open Science, Moscow, Russia) for 5 days a week. Immobilization is a traditional model of stress in which, in addition to movement restriction, there is a pronounced emotional component associated with the inability to avoid a threatening situation. Rats of the experimental group CCl_4_ received a standard semi-synthetic diet (lard and sunflower oil as fat components, 375 ± 3 kcal). On the 62nd day of the experiment, the animals were intraperitoneally injected with a 50% solution of CCl_4_ in olive oil at a dose of 1 mL/kg body weight [59,60,61,62].

On the 43rd day of the experiment, we studied body composition using a quantitative magnetic resonance system (EchoMRI LLC, Houston, TX, USA) that allows measuring total body fat mass, lean body mass, free water content and total body water volume of rats in less than 1 min in a living animal without anesthesia or euthanasia.

We tested animals in the elevated plus maze on the 40th day of the experiment to assess their level of anxiety and exploratory behavior during periods of their minimum daily activity (from 10:00 to 15:00). The movement of rats in the maze, the time spent in the open and closed arms, the number of zone transitions, and the distance traveled were recorded with the Smart 3.0.04 video tracking system (Panlab Harvard Apparatus, Barcelona, Spain).

Twenty-four hours prior to the end of the experiment, animals were placed in metabolic cages to collect daily urine. On day 63 of the experiment, we measured the fasting glucose level in blood taken from the tail vein using an electrochemical glucometer (OneTouch Select, Malvern, PA, USA).

On the 63rd day, rats from all groups (deprived for 12 h) were decapitated under light anesthesia, and a pathological autopsy was performed. One milliliter of blood was collected in test tubes with K_2_EDTA to determine glycated hemoglobin, the remaining blood collected after decapitation was incubated at 2–8 °C for 3 h, centrifuged for 30 min at 3000 rpm at 4 °C, and the resulting serum was stored at −20 °C. The liver was extracted by pathological dissection and weighed. A sample of liver (approximately 0.5 g) was collected to determine the expression of antioxidant enzymes; the remaining liver was additionally weighed, frozen, and lyophilized prior to fat extraction.

The content of glycated hemoglobin was determined spectrophotometrically using the commercial Glycohemotest Kit (ELTA, Moscow, Russia). The method is based on the principle of affinity separation of glycated and non-glycated fractions of hemoglobin of blood hemolysate on a sorbent with grafted 4-aminomethylphenylboronic acid.

Parameters of protein metabolism (total protein, albumin, globulins, urea, creatinine), lipid metabolism (total cholesterol, HDL cholesterol, LDL cholesterol, triglycerides), carbohydrate metabolism (glucose), purine metabolism (uric acid), mineral metabolism (calcium, phosphorus, magnesium), and liver function (total bilirubin, ALT, AST, alkaline phosphatase) were determined in blood serum using the automatic biochemical analyzer “Konelab 20i” (ThermoScientific, Waltham, MA, USA). Fat was extracted from the liver using the Folch method [63]. Samples of lyophilized liver were ground and transferred into test tubes. A mixture of chloroform and methanol (2:1) in a ratio of 1/10 was added to the samples. The samples were stirred for 1.5 h, then distilled water was added in a volume of 1/3 of the solvent volume, after which the mixture was centrifuged at 3500 rpm for 5 min. The fat dissolved in chloroform, which settled at the bottom of the test tube, was collected in pre-weighed flasks. After this, chloroform was added to the test tubes in a volume of 1/3 of the original volume of solvents, centrifuged at 3500 rpm for 5 min, and the fat dissolved in chloroform was again transferred into the flasks. The resulting combined fat extracts were evaporated on a rotary evaporator until the chloroform was completely removed. The flasks with fat were placed in a muffle furnace at 80 °C for 15 min. After cooling, the flasks were weighed and the mass of extracted fat was determined by the difference with the original flask mass. Before analysis, the fat sample was pre-dissolved in 95% ethanol at a concentration of 2.5 mg/mL. The content of triglycerides and cholesterol in fat extracted from the liver was determined photometrically using a Konelab 20i automatic biochemical analyzer (ThermoScientific, Waltham, MA, USA).

The catalase, glutathione peroxidase, and superoxide dismutase levels, as well as the degree of accumulation of lipid hydroperoxides and malondialdehyde (MDA), were determined in blood serum using the competitive ELISA analysis method according to the manufacturer’s instructions (Elabscience, Cloud Clone, Fine Test, Houston, TX, USA).

Sample preparation of daily urine included catecholamine derivatization (epinephrine, norepinephrine, and dopamine) in order to separate them under reverse-phase chromatography conditions and achieve the required ranges of quantitative determination. Carbonate buffer and benzoyl chloride solution in acetonitrile were added to urine samples, mixed and incubated at room temperature. Obtained solutions were then analyzed using HPLC-MS/MS.

Catecholamine levels in daily urine were determined by HPLC-MS/MS using an Agilent 1200 Series chromatograph (Agilent Technologies, Santa Clara, CA, USA) and a Thermo LTQ Velos Pro mass spectrometer (Thermo Fisher Scientific, Waltham, MA, USA). Separation of catecholamine derivatives in the water–acetonitrile system was carried out on an Agilent ZORBAX Eclipse XDB-C18 chromatographic column in gradient elution mode.

### 4.4. Antioxidant System Gene Expression

The expression of the following genes was assessed in rat liver using real-time reverse transcription polymerase chain reaction (RT-PCR): anti-inflammatory nuclear transcription factor (*NfkB*), transcription factor Nrf2 (*Nrf2*), superoxide dismutase (*Sod1*), glutathione peroxidase (*Gpx1*), catalase (*Cat*), NAD(P)H-quinone oxidoreductase (*Nqo1*), and heme oxygenase-1 (*Hmox1*). Total RNA was isolated from liver tissue using the ExtractRNA reagent (Eurogen, Moscow, Russia), and complementary DNA was synthesized using the MMLV RT kit (Eurogen, Moscow, Russia) according to the manufacturers’ protocol. For real-time PCR, primers and probes from DNA Synthesis LLC (Moscow, Russia) were used; the primer sequences are shown in Table 8.

The PCR reaction mixture with a total volume of 25 μL contained 2.5 μL of cDNA (diluted 1:10) obtained in the reverse transcription reaction from 2 μg of total RNA, 2.5 μL of 10X Taq Turbo buffer for HS Taq DNA polymerase (with 2.5 mM MgCl_2_) (Eurogen, Moscow, Russia), 1 μL of (F + R) primers (10 μM), 0.5 μL of FAM probe (10 μM), 1.0 μL of dNTPs mixture (10 mM) (Eurogen, Moscow, Russia), 0.25 μL of HS Taq DNA polymerase (5 U/mL) (Eurogen, Moscow, Russia), and 17.25 μL of nuclease-free water (Thermo Scientific, Waltham, MA, USA). Amplification was performed on a CFX 96 device (Bio-Rad, Hercules, CA, USA). Gene expression was assessed by the threshold cycle value and normalized relative to the reference genes *Actb* and *Gapdh* using the 2^−ΔΔCt^ method.

### 4.5. Statistical Analysis

We used the sample homogenous by sex, age, and line to reliably identify a significant biological response through statistical analysis.

Statistical processing of the obtained results was carried out using the SPSS Statistics 20 (IBM) software package. The sample was tested for normality using the Kolmogorov–Smirnov test at *p* = 0.05. In the case of normal distribution, parametric research methods were used: ANOVA according to plan 4^1^ (single-factor, four-level, balanced experiment). In case of rejection of the null hypothesis, the method of multiple comparison of means was used—Tukey’s criterion (q-test). The mean (M) and standard error of the mean (SEM) were calculated; data are presented as the M ± SEM. If the sample did not correspond to a normal distribution, nonparametric goodness-of-fit tests were used: Kruskal–Wallis test. In case of rejection of the null hypothesis, the multiple comparison method was used–Duncan’s test (MRT). The median (Me) and lower (Q1) and upper (Q3) quartiles were determined. Data are presented as Me (Q1–Q3). Differences were considered statistically significant at *p* < 0.05.

We calculated η^2^. The classification and interpretation of η^2^ (the strength of the effect) are presented in Table 9.

## 5. Conclusions

The combined consumption of sunflower oil, margarine, cholesterol, and fructose by animals resulted in the development of non-alcoholic fatty liver disease, characterized by an increase in the relative organ weight and extreme accumulation of fat, cholesterol, and triglycerides in the liver. In addition, a significant increase in the alanine aminotransferase serum concentration was revealed, accompanied by significant changes in the antioxidant status of rats: accumulation of serum MDA and activation of SOD. A significant increase in serum total cholesterol and LDL point at the development of hyperlipidemia in these animals.

Immobilization stress had a strong effect on the animals’ appetite, which led to a significant loss of their body weight; lean mass predominated in the body composition of these animals, with a significant decrease in the fat quota. The relative liver weight of these animals was significantly reduced against the background of weight loss; the fat content in the liver was also significantly lower. Immobilization had no effect on the liver levels of cholesterol and triglycerides. The blood biochemistry test showed a decrease in cholesterol level along with an increase in LDL and ALT. The effect of immobilization on the antioxidant system is shown only by a decrease in blood lipid peroxides.

Partial replacement of the diet lipid component with sunflower oil led to a significant decrease in animals’ appetite and a decrease in body weight, without changing the body composition, expressed in relative units. A single intraperitoneal injection of CCl_4_ against the background of sunflower oil consumption led to a significant increase in lipid peroxidation in the blood, characterized by the lipid peroxide accumulation and catalase activation. The significant increase in blood ALT and liver cholesterol shows the rapid development of toxic liver injury. In addition, a disturbance in carbohydrate metabolism was detected, expressed by a decrease in blood glucose and glycated hemoglobin.

The immobilization model has a serious impact on the psycho-emotional state of animals, which is accompanied by a loss of appetite, further leading to body exhaustion, a decrease in organ weight and changes in a number of biochemical parameters. However, in case of oxidative stress, it provides the least effect, resulting only in decreased levels of lipid peroxides. This model can be effectively used to evaluate the effects of adaptogens with anti-anxiety properties.

We have found that treating animals with a misbalanced diet containing sunflower oil, margarine, cholesterol, and fructose has the greatest impact on the rats’ bodies. The obtained data may be of interest in modeling hyperlipidemia; the model can be effectively used to reproduce non-alcoholic fatty liver disease in vivo and to assess the state of oxidative stress in the body of animals.

A single intraperitoneal administration of CCl_4_ against the background of a decrease in the omega-3 fatty acid quota in the animal’s body is accompanied by a change in a number of biochemical parameters characterizing oxidative stress, and can be effectively used to study the effects of antioxidants in an in vivo experiment.

## Figures and Tables

**Table 1:** Fatty acid composition of soybean and sunflower oil, %.

Fatty Acid (FA)	FA Index	Soybean Oil	Sunflower Oil
Myristic	14:0	0.10	0.07
Pentadecanoic	15:0	0.02	0.01
Palmitic	16:0	10.29	6.14
Hexadecene	16:1	0.02	0.02
Palmitoleic	16:1 9-cis	0.08	0.04
Margarine	17:0	0.08	0.03
Heptadecenoic	17:1	0.04	0.03
Stearic	18:0	4.92	3.16
Oleic	18:1 9-cis	24.11	27.31
Vaccenic	18:1 11-cis	1.40	0.78
Octadecene	18:1 11-cis	0.06	0.00
cis, trans-linoleic	18:2 9-cis, 12-trans	0.05	0.24
trans, cis-linoleic	18:2 9-trans, 12-cis	0.00	0.19
Linoleic	18:2	47.22	60.58
Octadecatrienic	Total trans-isomers	0.13	0.00
α-linolenic	18:3 ω-3	10.17	0.08
Arachidic	20:0	0.49	0.24
gondoic (total isomers)	20:1	0.24	0.20
Behenic	22:0	0.43	0.72

**Table 2:** Body composition of experimental animals.

Parameter	Group				Statistics	
	Control	DI	Immo	CCl_4_	*p*	η^2^
Fat mass, %	13.7 (11.8–15.9)	13.9 (9.7–15.2)	8.1 (6.7–11.7) ^1,2^	12.3 (8.7–15.5)	0.05	0.17
Lean mass, %	79.8 ± 1.0	80.8 ± 1.1	85.1 ± 1.1 ^1,2^	81.9 ± 1.5	0.02	0.20
Free water, %	0.25 (0.22–0.29)	0.20 (0.17–0.23) ^1^	0.32 (0.29–0.35) ^1,2^	0.21 (0.17–0.23) ^1,3^	<0.01	0.39
Total water, %	67.8 ± 0.9	68.5 ± 0.9	72.3 ± 0.9 ^1,2^	69.7 ± 1.2	0.02	0.21

^1^ differences are significant compared to control group; ^2^ differences are significant compared to DI group; ^3^ differences are significant compared to Immo group; *p* ≤ 0.05.

**Table 3:** Biochemistry blood test.

Parameter	Animal Groups				Statistics	
	Control	DI	Immo	CCl_4_	*p*	η^2^
Total protein, g/L	71.9 ± 1.4	77.4 ± 1.8 ^1^	71.5 ± 1.1 ^2^	72.7 ± 1.5 ^2^	0.02	0.20
Albumin, g/L	30.5 ± 0.3	31.5 ± 0.5	30.6 ± 0.3	30.3 ± 0.4 ^2^	0.04	0.12
Globulins, g/L	41.0 [38.0–44.0]	44.8 [43.4–49.2] ^1^	40.1 [38.9–43.3] ^2^	41.8 [39.9–43.8]	0.04	0.21
Cholesterol, mmol/L	1.65 [1.37–1.79]	2.52 [1.83–2.79] ^1^	1.51 [1.38–1.76] ^2^	1.11 [0.96–1.47] ^1,2,3^	<0.01	0.49
Triglycerides, mmol/L	2.95 [2.29–3.86]	2.17 [1.38–2.85] ^1^	1.36 [1.02–1.69] ^1^	0.74 [0.53–1.07] ^1^	<0.01	0.44
HDL, mmol/L	1.28 ± 0.09	1.33 ± 0.09	1.41 ± 0.05	1.10 ± 0.09 ^2,3^	0.05	0.17
LDL, mmol/L	0.10 [0.09–0.11]	0.44 [0.33–0.70] ^1^	0.15 [0.13–0.17] ^1,2^	0.08 [0.07–0.12] ^2,3^	<0.01	0.65
Total bilirubin, μmol/L	5.01 [4.79–5.46]	4.51 [4.36–5.38]	5.16 [5.00–5.43] ^2^	5.30 [5.00–5.62] ^2^	0.03	0.10
Creatinine, μmol/L	46.3 ± 0.8	46.3 ± 1.3	49.0 ± 0.8	46.3 ± 0.9	0.12	0.12
Alkaline phosphatase, U/L	159 [143–213]	191 [178–202]	154 [139–202]	144 [137–171] ^2^	0.02	0.15
ALT, U/L	74.3 [60.9–84.4]	91.2 [72.5–148.1] ^1^	89.5 [83.5–101.1] ^1^	102.7 [79.0–109.7] ^1^	0.02	0.17
AST, U/L	40.0 [34.2–172.8]	201.7 [52.2–230.7]	239.4 [81.7–258.7] ^1^	62.5 [42.9–271.3]	0.03	0.04
Urea, mmol/L	6.33 ± 0.24	4.08 ± 0.29 ^1^	5.59 ± 0.28 ^2^	6.70 ± 0.36 ^2,3^	<0.01	0.51
Uric acid, mmol/L	85.1 [81.3–92.8]	95.7 [81.6–107.1]	90.4 [87.1–103.5]	77.5 [73.3–93.9]	0.31	0.05
Phosphor, mmol/L	2.12 [2.04–2.74]	2.07 [1.91–2.32]	2.39 [2.22–2.53] ^2^	2.39 [2.35–2.54] ^2^	0.03	0.15
Magnesium, mmol/L	0.90 [0.78–0.99]	0.73 [0.65–0.77] ^1^	0.85 [0.82–1.02] ^2^	0.79 [0.76–0.83]	<0.01	0.26
Calcium, mmol/L	2.45 [2.31–3.36]	2.42 [2.40–2.61]	2.38 [2.31–2.42]	2.45 [2.38–2.50]	0.51	0.04
Glucose, mmol/L	6.78 [5.91–7.19]	7.87 [7.63–8.25] ^1^	6.10 [5.65–6.47] ^2^	5.93 [5.84–6.27] ^1,2^	<0.01	0.43
Glucose, mmol/L (whole blood)	5.85 [5.30–6.28]	7.30 [7.05–7.55] ^1^	5.55 [5.18–6.33] ^2^	5.95 [5.53–6.03] ^2^	<0.01	0.53
Glycated hemoglobin, %	6.3 [5.4–6.8]	6.7 [5.7–7.5]	6.4 [5.9–7.8]	5.2 [5.0–5.4] ^1,2,3^	0.03	0.05

^1^ differences are significant against control group; ^2^ differences are significant against DI group; ^3^ differences are significant against Immo group; *p* < 0.05.

**Table 4:** Liver biochemical parameters.

Parameter	Animal Groups				Statistics	
	Control	DI	Immo	CCl_4_	*p*	η^2^
Final body weight, g	479 [440–505]	449 [438–498]	369 [360–392] ^1,2^	430 [404–448] ^1,3^	<0.01	0.38
Liver weight, relative, %	2.72 [2.60–2.93]	4.33 [4.08–4.69] ^1^	2.42 [2.27–2.53] ^1,2^	2.68 [2.57–2.76] ^2,3^	<0.01	0.87
Fat content, mg/g liver	89.4 [81.4–114.2]	189.6 [121.4–223.5] ^1^	76.3 [63.3–83.3] ^1,2^	93.9 [85.6–105.5] ^2,3^	<0.01	0.49
Cholesterol, mg/g liver	5.79 [4.14–6.92]	14.2 [6.1–16.9] ^1^	4.12 [3.38–5.19] ^2^	17.3 [12.8–18.5] ^1,3^	<0.01	0.62
Triglycerides, mg/g liver	44.0 [34.7–59.2]	64.2 [48.6–98.4] ^1^	38.2 [32.0–42.2] ^2^	33.3 [24.7–38.7] ^1,2^	0.01	0.31

^1^ differences are significant against control group; ^2^ differences are significant against DI group; ^3^ differences are significant against Immo group; *p* < 0.01.

**Table 5:** Blood serum biochemical parameters determined by ELISA.

Parameters	Animal Groups				Statistics	
	Control	DI	Immo	CCl_4_	*p*	η^2^
Lipid peroxides, ng/mL	362.8 [318.9–438.5]	381.4 [345.3–544.4]	258.6 [246.2–291.1] ^1^	482.8 [420.0–534.5] ^1^	<0.01	0.31
MDA, ng/mL	190.4 [149.8–233.1]	352.4 [226.0–462.2] ^1^	191.5 [146.2–295.8]	168.3 [120.6–218.9] ^2^	0.05	0.13
Glutathione peroxidase, pg/mL	69.5 [55.5–82.1]	67.7 [58.2–72.5]	69.7 [55.6–87.2]	60.0 [49.9–81.2]	0.88	0.07
Catalase, ng/mL	1.42 [1.03–1.61]	1.58 [1.28–1.93]	1.03 [0.83–1.29] ^2^	2.34 [1.81–2.44] ^1,3^	<0.01	0.20
SOD, ng/mL	2.75 [2.31–3.07]	6.55 [3.91–9.78] ^1^	4.65 [2.20–6.46]	2.46 [1.78–3,52] ^2^	0.037	0.20

^1^ differences are significant compared to control group, ^2^ differences are significant compared to DI group, ^3^ differences are significant compared to Immo group; *p* ≤ 0.05.

**Table 6:** Antioxidant gene and transcription factor expression in liver tissues.

Gene	Animal Group				Statistics	
	Control	DI	Immo	CCl_4_	*p*	η^2^
*Cat*	1.04 [0.90–1.12]	0.68 [0.66–0.80] ^1^	0.96 [0.94–1.11] ^2^	0.70 [0.61–0.79] ^1,3^	<0.01	0.30
*Sod1*	0.98 [0.91–1.09]	0.77 [0.71–0.92]	1.13 [1.04–1.17] ^2^	1.17 [1.01–1.51] ^2^	0.02	0.53
*Gpx1*	1.04 [0.89–1.19]	0.83 [0.69–0.86]	0.76 [0.70–0.91]	0.54 [0.49–0.59] ^1,3^	<0.01	0.44
*Hmox1*	0.94 [0.82–1.11]	1.05 [0.86–1.32]	0.64 [0.52–0.80]	0.80 [0.58–1.17]	0.34	0.18
*Nqo1*	1.36 [0.85–1.57]	1.61 [1.14–3.21]	1.64 [0.96–2.75]	7.13 [5.05–9.53] ^1,2,3^	<0.01	0.44
*Nrf2*	1.06 [1.05–1.16]	0.96 [0.82–1.16]	1.07 [0.97–1.18]	0.87 [0.73–1.03]	0.43	0.18
*Nfkb1*	1.04 [0.95–1.95]	0.95 [0.84–1.03]	0.94 [0.90–1.01]	1.05 [1.00–1.12]	0.62	0.14

^1^ differences are significant against control group; ^2^ differences are significant against DI group; ^3^ differences are significant against Immo group (*p* < 0.05).

**Table 7:** Animal groups.

Parameter	Animal Group			
	Control	DI	Immo	CCl_4_
Body weight, g	157 ± 3	160 ± 3	160 ± 3	157 ± 3
Open Field Test Results				
Time in center, s	1.4 ± 0.6	1.1 ± 0.5	1.9 ± 0.5	1.7 ± 0.8
Zone transitions	5.8 ± 1.0	5.5 ± 1.3	6.2 ± 0.9	5.7 ± 1.1
Total distance, cm	1581 ± 135	1729 ± 109	1768 ± 100	1741 ± 119

**Table 8:** The primer sequences.

Primer	Sequence
*NfkB*	F CGTGGAGTACGACAACATCTC
	R GAGGTGTCGTCCCATCGTA
	FAM CTGCTCCTGGAGGGTGACGC-BHQ1
*Nrf2*	F CACATCCAGACAGACACCAGT
	R GAATGTCTCTGCCAAAAGC
	FAM CTCCCAGGTTGCCCACATTCCC-BHQ1
*Hmox1*	F CCAGCCTGAACTAGCCCA
	R CCTTGGTGGCCTCCTTC
	FAM CCACAGCTCGACAGCATGTCC-BHQ1
*Sod1*	F GACAATACACAAGGCTGTACCA
	R CAGGTCTCCAACATGCCTC
	FAM CTCACTCTAAGAAACATGGCGGTC-BHQ1
*Cat*	F GTCTGGGACTTCTGGAGTCTT
	R CATAGCCATTCATGTGCCG
	FAM CCATCAGGTTACTTTCTTGTTCAG-BHQ1
*Gpx1*	F TCAGTTCGGACATCAGGAGA
	R CATTCACCTCGCACTTCTCAA
	FAM CCCTCAAGTATGTCCGACCCG-BHQ1
*Nqo1*	F GGGACATGAACGTCATTCTC
	R CACCAGTTGAGGTTCTAAGACC
	FAM CAATTCAGAGTGGCATTCTGCGC-BHQ1

**Table 9:** Classification of the effect strength for η^2^.

Effect Strength	Value of η^2^	Interpretation
Weak	0.01–0.06	The factor explains only a small part of the variation in the dependent variable. The effect may be statistically significant with a large sample size.
Average	0.06–0.14	The factor explains a moderate part of the variation in the dependent variable. The effect can be practically significant.
Strong	≥0.14	The factor explains a significant part of the variance in the dependent variable. The effect is likely to have practical meaning.

## Data Availability

The data presented in this study are available on request from the corresponding author.

## Abbreviations

The following abbreviations are used in this manuscript:

ALT	Alanine aminotransferase
ALP	Alkaline phosphatase
AST	Aspartate aminotransferase
HDL	High-density lipoprotein
LDL	Low-density lipoprotein
MDA	Malon dialdehyde
OF	Open Field
PCR	Polymerase chain reaction
ROS	Reactive oxygen species
SOD	Superoxide dismutase
